# Persistent Postsurgical Pain in Oral Cancer Patients Reconstructed with Anterolateral Thigh Free Flap

**DOI:** 10.3390/medicina58030391

**Published:** 2022-03-06

**Authors:** Ya-Chun Shen, Kuei-Lin Liao, Kuang-I Cheng, Kuang-Yi Tseng, Miao-Pei Su

**Affiliations:** 1 Department of Anesthesiology, Kaohsiung Medical University Hospital, Kaohsiung 80756, Taiwan; adam880725@yahoo.com.tw (Y.-C.S.); mn103540004@gmail.com (K.-L.L.); kuaich@gmail.com (K.-I.C.); deepbluetseng@gmail.com (K.-Y.T.); 2 School of Medicine, College of Medicine, Kaohsiung Medical University, Kaohsiung 80756, Taiwan

**Keywords:** postsurgical pain, oral cancer, anterolateral flap, quality of life

## Abstract

*Background and Objectives*: The anterolateral thigh (ALT) flap is widely used in head and neck reconstruction, but the postoperative thigh sensory function lacks sufficient evaluation. The present study reports the postsurgical pain and cancer-related quality of life (QoL) in different stages of oral cancer patients receiving anterolateral thigh (ALT) flap reconstruction. *Materials and Methods*: Patients were subgrouped into postoperative early-, mid-, and late-recovery stages (postoperative 0.5–1 years, 1–2 years, and above 2 years) according to the time point of assessment. The QoL was examined using the EORTC C-30. Postsurgical donor and receipt site pain was evaluated through subjective reports and sensory tests. *Results*: Ninety-four patients were included in the final analysis. The functional and global health-related QoL significantly improved with time after surgery. However, spontaneous pain was reported in 57.7%, 72.3%, and 42% of patients in early-, mid-, and late-recovery stages, mainly in donor sites rather than in receipt sites. The highest incidence of donor site pain after ALT flap reconstruction in oral cancer surgery was in the mid-recovery stage but remained high in the late-recovery stage (56.8% and 36.7%, respectively). *Conclusions*: The postsurgical pain in the donor site might persist to or exhibit delayed onset one to two years postoperatively but is much improved after postoperatively two years later. A longer postsurgical follow-up for over two years for pain and sensory dysfunction is indicated.

## 1. Introduction

Immediate flap reconstruction for oral or maxillofacial defects resulting from radical resection for oral cancer is often needed. The type of reconstruction, type of surgery, and cancer stage may influence the postoperative quality of life (QoL) [1,2,3].

The anterolateral thigh (ALT) free flap is widely used in reconstructive surgery for head and neck cancer for the advantages of versatile flap designs and minimal donor site morbidity [4,5]. Early and late donor site complications of ALT flap, although rare, including compartment syndrome, muscle necrosis, muscle herniation, hemangioma and neuroma formation, and donor limb weakness have been reported and discussed [6]. However, Townley et al. [7] have reported a donor site assessment after ALT flap reconstruction, showing persistent pain in 15% of patients at postoperative six months. Weise et al. [8] identified 82.4% of patients with hypesthesia at the donor site in a variable follow-up duration. However, the characteristics of pain, a quantitative evaluation of thigh sensory function at the donor sites, and the influence on postoperative QoL lacked sufficient evaluation, especially in the long-term follow-up. In a retrospective review [9], head and neck cancer pain can lead to chronic opioid use, which is associated with decreasing survival.

The purpose of this study is to investigate the characteristics and incidence of persistent postsurgical pain in oral cancer patients receiving ALT flap reconstruction, and the relationship with cancer-related QoL at different postoperative stages.

## 2. Materials and Methods

This study was approved by the hospital’s Institutional Review Board (KMUH-IRB-20130085) and registered at ClinicalTrials.gov Identifier (NCT02048631). Between July 2013 and February 2014, 357 consecutive adult oral cancer patients who underwent wide excision of oral tumors reconstructed with ALT free flaps were selected in the outpatient department of plastic surgery at Kaohsiung Medical University Hospital, a tertiary medical center. Patients who were older than 70 years of age, impaired in communication, diagnosed with dementia and lacked the ability to complete the questionnaire by themselves were excluded. Written informed consent was obtained from each patient before enrollment in the study.

According to the time interval between surgery and evaluation, patients were categorized into the postoperative early-recovery stage (postoperative six months to one year), mid-recovery stage (one to two years), and late-recovery stage (over two years).

### 2.1. General Anesthesia and Operative Procedures

Patients who underwent free flap surgery received inhalational general anesthesia with nasotracheal intubation. Sevoflurane was used for the maintenance of anesthesia. The operation procedure is briefly described as follows. A wide excision was planned for the tumors and the surrounding tissues, preparing a clear border for free flap reconstruction of facial defects. Several procedures were applied to establish a well-prepared free flap for reconstruction: a hand-held Doppler probe was used to map the suitable perforators in the selected thigh tissue and design a flap according to the size and shape of the defect. The main axis of the ALT flap was marked by a line drawn from the anterior iliac spine to the lateral aspect of the patella. The pedicle of the ALT flap was supplied by the perforator from the descending branch of the lateral circumflex femoral vessels and the flap was centered on the chosen perforators along the main longitudinal axis. The chosen ALT perforators were dissected along the subfascial plane and its whole length muscles were free. Finally, the thin fascial ALT flap was harvested just within the fascia and a small amount of overlying fat. The flap design could be adjusted depending on the findings of a Doppler test. Flap donor areas wider than 8 cm were considered for skin grafts. The donor site was closed directly or received skin grafting with a split-thickness skin graft or a full-thickness skin graft.

### 2.2. Measurement of Donor and Receipt Sites Postsurgical Pain Characters

In the plastic surgery outpatient department for postoperative assessment, all patients were requested to complete a chronic pain-related questionnaire by themselves. Patients were asked to report postsurgical donor and receipt site pain with a numeric rating scale (NRS: 0—no pain and 10—worst pain imaginable), pain locations, self-reported spontaneous pain (tingling, burning, aching, electric shocks or shooting, and twitch pain) with continuous or paroxysmal symptoms, and dysesthesia. Subsequently, stimulus-evoked positive sensory responses were recorded by a registered nurse anesthetist from the contralateral limb to the donor limb, lateral to medial sides, and outer border to the inner part of the surgical sites. Brush and cotton wool were used for light touch-induced pain. Mechanical static pressure-induced pain was measured using 10 g and 2 g von Frey monofilaments (Stoelting, Wood Dale, IL, USA) on the donor sites and contralateral thigh alternately, to differentiate the mechanical dysesthesia. Each filament was steadily compressed perpendicular to the skin until filament bending occurred. A pinprick wheel roller (Wartenberg pinwheel, Poulsbo, Washington, DC, USA) was used for pin-pricking-induced pain. Metal temperature rollers, one warm at 40 °C and the other cold at 25 °C (SENSELab ROLLTEMP, Somedic, Sweden), were used for screening temperature sensibility. The negative phenomena included numbness or hypoalgesia on stimuli-evoked sensory responses on donor and receipt sites.

### 2.3. Assessment of Health-Related Cancer Quality of Life

The European Organization for Research and Treatment of Cancer Quality of Life Questionnaire (Core-30 version 3 in Chinese, EORTC QLQ C-30) was used to measure the quality of life for cancer patients. Each patient was asked to complete the core questionnaire sections that incorporate physical, emotional, and social health issues with multi-item scales, including five functional scales, three symptom scales, a global health status, and six single-item scales. Score calculations are based on the Fayers et al. scoring procedures [10] by first calculating the raw scores and then applying the linear transformation to obtain functional scores, symptoms/problem scores, and global health status with assigned formulas. Higher functional or global health status scores indicate a higher (more positive) level of functioning or global health-related quality of life. A high score for a symptom scale/item represents a high level of symptomatology/problems.

### 2.4. Statistical Analysis

Statistical analyses were performed using SPSS software (Version 19.0.; Armonk, NY, USA: IBM Corp.). The chi-square test was used for the comparison of sensory test results between the donor and contralateral sites, and spontaneous pain characteristics between different postoperative stages. The pain intensity NRS was analyzed with one-way ANOVA. The EORTC average scores were compared for each domain using one-way ANOVA. The significance level was set at *p* < 0.05.

## 3. Results

**Figure 1:**
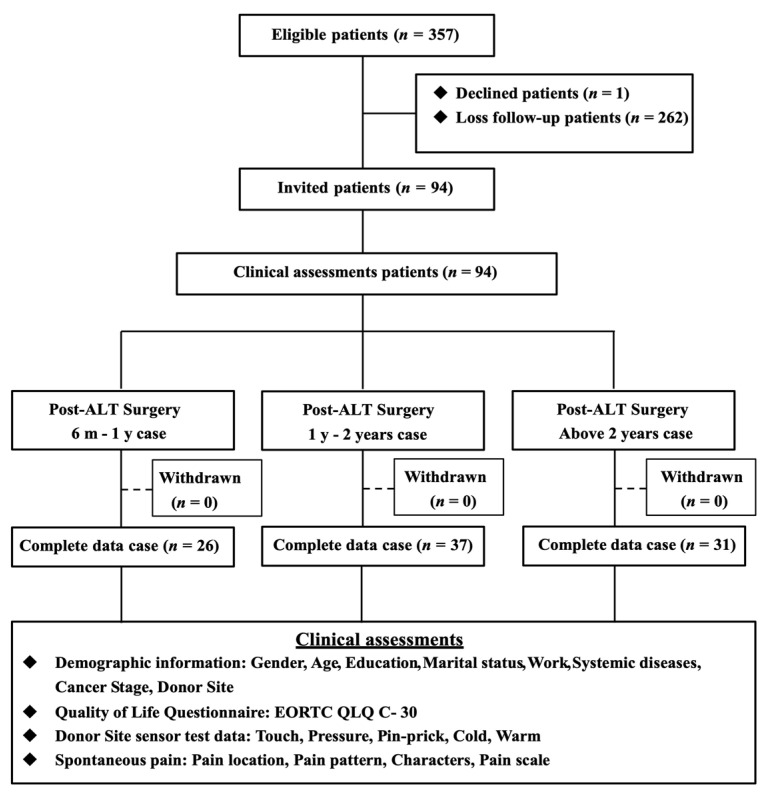
The study protocol. Abbreviations: ALT, anterolateral thigh.

The study protocol is summarized in Figure 1.

### 3.1. Patient Characteristics

Nighty-four patients were enrolled in the final analysis. Most of the patients were in their fifties, and males accounted for more than 90% of the subjects in all three groups (Table 1). The education, marital status, employment status, presence of systemic diseases, and cancer stage were not significantly different between groups. Stage 4 cancer remained in the majority in our study (above 60% in all groups). Most of the patients received a single ALT flap, while one patient in the early-recovery stage group and two patients in the late-recovery stage group were reconstructed with double ALT flaps.

### 3.2. Cancer-Related Quality of Life Assessment

In the EORTC C-30 assessment, the patients in the postoperative late-recovery stage (>2 years) had the highest scores for global health status, representing the highest quality of life, followed by the patients in the mid-recovery stage (postoperative 1–2 years) and the early-recovery stage (6 months–1 year) after surgery (Table 2). The role and social function were also significantly better in patients in the late-recovery stage, while the physical, emotional, and cognitive functions were not significantly different between stages. In the symptom/problem items, the level of pain, financial difficulty, nausea/vomiting, constipation, dyspnea, diarrhea and appetite loss were not statistically different between stages. Only insomnia and fatigue domains showed lower symptom levels in the postoperative late-recovery stage patients, followed by mid- and early-recovery stage patients.

### 3.3. Sensory Test

In the mechanical and thermal sensory tests, no patient demonstrated sensory dysfunction (an abnormal sensory test result) in the contralateral thigh. The proportion of sensory dysfunction present in the thigh donor site was significantly high in all three stages and all examined items, including light touch, pressure, pin-prick, and a cold and warm test (Table 3). The sensory dysfunction had a tendency to be located on the lateral aspect of the thigh donor site more than on the medial aspect. In the abnormal sensory test results, hyposensitivity, rather than hypersensitivity, was predominant for both mechanical and thermal stimuli (Table 4).

### 3.4. Patient-Reported Spontaneous Pain Characteristics

Nearly half of the patients reported spontaneous pain after ALT flap reconstruction for oral cancer surgery in all three stages, which was mainly located in the donor sites (>80%) than in the receipt sites (Table 5). The pain pattern was intermittent, and the most frequently reported pain characteristic was paresthesia, followed by numb pain and twitch. The ratio of patients seeking treatment for pain and the mean NRS values were both significantly lower in the late-recovery stage patients (Table 6). The ratios of patients with moderate pain (NRS 4 to 6) were higher in the early- and mid-recovery stages (19.2% and 24.1%, respectively), than in the late-recovery stage (3.2%), although there was no statistical difference.

## 4. Discussion

The ALT flap is the mainstream in our institute for oral cancer reconstruction because of the advantages of a long vascular pedicle, large skin territory and less donor site morbidity. However, donor site pain has been frequently reported in postoperative patients in our plastic surgery outpatient department. In our study, the global health status in the EORTC-C30 assessment gradually improved with time, although the prevalence of spontaneous pain was highest in the postoperative mid-recovery stage. This revealed an inconsistent trend between the pain and the cancer-related quality of life, which reminded us to evaluate these two components separately in postoperative follow-up.

Townley et al. [7] reported a 6-month follow-up in the ALT flap donor site, which revealed persistent pain in 15% of patients and numbness or tingling in 59% of patients. Weise et al. [8] revealed that 82% of patients had hypesthesia in the donor site during a sensory examination performed at least 6 months postoperatively (range 6–91 months). One systemic review [11] reported that pain lasting longer than 6 months affected 2.6% of patients receiving ALT flap harvesting. In our study, spontaneous pain remained at high prevalence in the postoperative early- and mid-recovery stage (6 months–1 year, 57.7%; 1–2 years, 72.3%), but showed a trend toward decreased prevalence in the late-recovery stage (>2 years, 42%). The proportion and duration of donor site pain and sensory dysfunction after ALT flap reconstruction was higher and longer than in early reports. The flap size, the need for skin grafting, and the patient’s expectation might be related to the difference. Further studies with a larger sample size and longer postsurgical follow-up may be indicted.

The pain intensity and the proportion of patients with moderate pain were highest in the postoperative mid-recovery stage (1–2 years). Although high-intensity acute postoperative pain is one of the main risk factors for persistent postsurgical pain, a delayed onset of persistent postsurgical pain has been observed in several studies [12,13,14]. One possible reason is the immediate postsurgical “honey-moon period” [15]. The beneficial effect of surgery and patients’ expectations of improvement may dominate and conceal the long-term consequences [16,17]. Secondly, nerve damage-associated neuropathic pain symptoms may be a delayed onset due to axonal sprouting or central sensitization [15,18]. The lateral femoral cutaneous nerve injury or sacrifice in ALT flap harvesting is a rational explanation, especially in large flap designs for advanced tumor stages. The hyposensitivity to stimuli in donor sites may also indicate a neuropathic pain component. However, in our survey, the pain intensity and the prevalence of neuropathic pain in donor sites were much lower after 2 years postoperatively, indicating the full recovery phase. Further studies related to the recovery from persistent postsurgical pain are needed.

In the EORTC-C30 assessment, our data showed a significant improvement in postoperative QoL over time in global health status, role functioning, social functioning, and symptoms of insomnia and fatigue. This trend is different from previous research. In a 2-year QoL study on head and neck reconstruction using the radial forearm flap, fibula flap, and scapular flap, the global QoL remained stable before and at 6 and 12 months after surgery [1]. ALT flap reconstruction may contribute to the QoL in patients with oral cancer treated with surgery, but further flap type-specific prospective studies are needed for stronger evidence of the changes in health-related QoL in different postoperative stages.

This study has some limitations. First, only the patients followed up in the plastic surgery department were enrolled in our study. In our institute, oral cancer surgery with an immediate ALT flap reconstruction is performed by three medical teams: the otolaryngology team or oral and maxillofacial surgery team for cancer tumor resection, and the plastic surgery team for flap reconstruction. Thus, outpatient tracing could happen in these three separate departments. Bias may occur in patient selection. Second, multiple factors are associated with postoperative QoL after ALT flap reconstruction for oral cancer surgery, including age, gender, radiotherapy, tumor location, and cancer stage [19,20,21]. In our study, concomitant medical treatments, such as radiotherapy and chemotherapy were not documented. Third, the pain intensity on NRS evaluation showed a significant decline in postoperative 2 years, but the symptom domain of pain in the EORTC-C30 assessment was not statistically different between the three stages. This diversity may exist because persistent postsurgical pain may not seriously influence patients’ daily activities, or because of the relatively small number of cases in our study.

Our study demonstrates a thorough survey of persistent postsurgical pain in oral cancer patients receiving ALT flap reconstruction. The overall cancer-related quality of life in these patients significantly improved with time after the operation. However, the prevalence of spontaneous pain at the donor site remained high and even developed one year after surgery. Therefore, a longer postsurgical follow-up for at least over two years for donor site pain and sensory dysfunction after ALT flap reconstruction in oral cancer patients is indicated.

## Figures and Tables

**Table 1:** Demographic data of patients included in each group following anterolateral thigh flap surgery.

Postoperative Stages	Early (6 m–1 y) (*n* = 26)	Mid (1–2 y) (*n* = 37)	Late (>2 y) (*n* = 31)	*p* Value
Gender				
Male	24 (92.3%)	37 (100%)	29 (93.5%)	0.251
Female	2 (7.7%)	0 (0%)	29 (6.5%)	
Age, y/o #	54.7 [51.1–58.3]	56.3 [53.4–59.3]	55.4 [52.4–58.4]	0.764
Education				
Elementary	13 (50.0%)	13 (35.1%)	13 (42.0%)	0.701
Junior	7 (26.9%)	12 (32.4%)	7 (22.6%)	
Senior or above	6 (23.1%)	12 (32.4%)	11 (35.5%)	
Marital status				
Married living together	16 (61.5%)	29 (78.4%)	25 (80.6%)	0.323
Divorced/Widowed/ Never married	10 (38.5%)	8 (21.6%)	6 (19.4%)	
Work				
Out of work/Retired	18 (69.2%)	23 (62.2%)	18 (58.1%)	0.779
Employed	8 (30.8%)	14 (37.8%)	13 (41.9%)	
Systemic diseases				
None	16 (61.5%)	23 (62.2%)	23 (74.2%)	0.888
Diabetes	2 (7.7%)	3 (8.1%)	2 (6.5%)	
Hypertension	6 (23.1%)	7 (18.9%)	3 (9.7%)	
Diabetes and hypertension	2 (7.7%)	4 (10.8%)	3 (9.7%)	
Cancer Stage				
Stage Ⅰ	2 (7.7%)	5 (13.5%)	2 (6.7%)	0.689
Stage Ⅱ	1 (3.8%)	2 (5.4%)	4 (13.3%)	
Stage Ⅲ	5 (19.2%)	4 (10.8%)	5 (16.7%)	
Stage Ⅳ	18 (69.2%)	26 (70.3%)	19 (63.3%)	
Donor Site				
Single ALT	25 (96.2%)	35 (100%)	29 (93.6%)	0.331
Double ALT	1 (3.8%)	0 (0%)	2 (6.4%)	

# Value is shown of the median with 95% confidence interval. Abbreviations: ALT, anterolateral thigh.

**Table 2:** Assessment of patients’ quality of life with the European Organization for Research and Treatment of Cancer Quality of Life Questionnaire C-30 (EORTC QLQ-C-30) following anterolateral thigh flap surgery. SD: standard deviation.

Content Area (Scale)	Items	Early (6 m–1 y) (*n* = 26)	Mid (1–2 y) (*n* = 37)	Late (>2 y) (*n* = 31)	*p* Value
		Mean (SD)	Mean (SD)	Mean (SD)	
Global health status ^(1)^	(29,30)	53.5 (21.4)	63.5 (19.8)	70.4 (18.0)	0.007 *
Functional ^(1)^					
Role functioning	(6,7)	76.3 (29.9)	86.9 (16.3)	90.3 (13.5)	0.030 *
Physical functioning	(1~5)	80.8 (18.2)	82.2 (14.8)	86.9 (12.7)	0.272
Social functioning	(26,27)	69.9 (25.4)	79.3 (20.6)	87.1 (23.1)	0.021 *
Emotional functioning	(21~24)	80.8 (16.5)	84.0 (16.8)	86.6 (14.7)	0.402
Cognitive functioning	(20,25)	78.2 (23.5)	79.7 (15.3)	84.4 (16.6)	0.399
Symptom/problem items ^(2)^					
Pain	(9,19)	22.4 (28.3)	18.5 (18.8)	10.2 (15.9)	0.082
Insomnia	(11)	41.0 (34.4)	27.0 (31.3)	19.4 (28.3)	0.036 *
Financial difficulties	(28)	32.1 (37.1)	33.3 (35.1)	28.0 (32.3)	0.809
Fatigue	(10,12,18)	25.2 (23.0)	15.6 (17.3)	6.5 (11.7)	<0.001 *
Nausea and Vomiting	(14,15)	0 (0.0)	3.2 (10.3)	0 (0.00)	0.075
Constipation	(16)	14.0 (25.3)	11.7 (23.9)	5.4 (19.4)	0.323
Dyspnea	(8)	10.3 (18.3)	11.7 (19.6)	7.5 (14.2)	0.619
Diarrhea	(17)	3.8 (10.9)	1.8 (7.6)	4.3 (11.4)	0.542
Appetite loss	(13)	7.7 (21.7)	6.3 (17.3)	3.2 (13.2)	0.607

*: *p* < 0.05. (1): A higher score indicating a higher (i.e., more positive) level of functioning or global health-related quality of life. (2): A higher (i.e., more negative) level of symptoms or problems.

**Table 3:** Different stimulus responses either on the medial and lateral donor or contralateral thigh skins following anterolateral thigh flap surgery.

Variables	Early (6 m–1 y) (*n* = 26)				*p* Value	Mid (1–2 y) (*n* = 37)				*p* Value	Late (>2 y) (*n* = 31)				*p* Value
	Contralateral		Donor			Contralateral		Donor			Contralateral		Donor		
	Normal	Abnormal	Normal	Abnormal		Normal	Abnormal	Normal	Abnormal		Normal	Abnormal	Normal	Abnormal	
Medial thigh portion															
Touch	26	0	15	11	<0.001 *	37	0	17	20	<0.001 *	31	0	15	16	<0.001 *
															0.641 ^#^
Pressure	26	0	13	13	<0.001 *	37	0	13	24	<0.001 *	31	0	10	21	<0.001 *
															0.343 ^#^
Pin-prick	26	0	14	11	<0.001 *	37	0	14	23	<0.001 *	31	0	7	24	<0.001 *
															0.052 ^#^
Cold	26	0	14	12	<0.001 *	37	0	17	20	<0.001 *	31	0	12	19	<0.001 *
															0.520 ^#^
Warm	26	0	14	12	<0.001 *	37	0	19	18	<0.001 *	31	0	12	19	<0.001 *
															0.451 ^#^
Lateral thigh portion															
Touch	26	0	12	14	<0.001 *	37	0	16	21	<0.001 *	31	0	17	14	<0.001 *
															0.621 ^#^
Pressure	26	0	7	19	<0.001 *	37	0	10	27	<0.001 *	31	0	10	21	<0.001 *
															0.868 ^#^
Pin-prick	26	0	5	21	<0.001 *	37	0	6	31	<0.001 *	31	0	9	22	<0.001 *
					0.007 ^&^					0.036 ^&^					0.418 ^#^
Cold	26	0	6	20	<0.001 *	37	0	8	29	< 0.001 *	31	0	10	21	<0.001 *
					0.023 ^&^					0.027 ^&^					0.572 ^#^
Warm	26	0	6	20	<0.001 *	37	0	11	26	<0.001 *	31	0	8	23	<0.001 *
					0.023 ^&^					0.058 ^&^					0.310 ^#^

* the value revealed contralateral and donor site comparisons in each group in the same pain character; &: the value revealed patient number comparisons in each group between the medial and lateral thigh in the same pain character; # the value revealed donor sites comparisons among groups.

**Table 4:** Abnormal senses with different stimuli on the medial and lateral donor thigh skin following anterolateral thigh flap surgery.

Variables	(N)	Early (6 m–1 y)		(N)	Mid (1–2 y)		(N)	Late (>2 y)	
		Positive	Negative		Positive	Negative		Positive	Negative
Medial thigh portion									
Touch	(*n* = 11)	1	10	(*n* = 20)	6	14	(*n* = 16)	1	15
Pressure	(*n* = 13)	2	11	(*n* = 24)	6	18	(*n* = 21)	1	20
Pin-prick	(*n* = 12)	4	8	(*n* = 23)	10	13	(*n* = 24)	9	15
Cold	(*n* = 12)	3	9	(*n* = 20)	3	17	(*n* = 19)	4	15
Warm	(*n* = 12)	0	12	(*n* = 18)	1	17	(*n* = 19)	5	14
Lateral thigh portion									
Touch	(*n* = 14)	1	13	(*n* = 21)	1	20	(*n* = 14)	1	13
Pressure	(*n* = 19)	2	17	(*n* = 27)	0	27	(*n* = 20)	1	20
Pin-prick	(*n* = 21)	5	16	(*n* = 31)	5	26	(*n* = 22)	8	14
Cold	(*n* = 20)	2	18	(*n* = 29)	2	27	(*n* = 21)	1	20
Warm	(*n* = 20)	1	19	(*n* = 26)	0	26	(*n* = 23)	1	22

Positive: increased responses; negative: decreased responses.

**Table 5:** Pain characteristics at each time interval following anterolateral thigh flap surgery.

Variables	Early (6 m–1 y) (*n* = 26)	Mid (1–2 y) (*n* = 37)	Late (>2 y) (*n* = 31)	*p* Value
Spontaneous Pain				
No	11 (42.3%)	11 (29.7%) *	18 (58%) *	0.061
Yes	15 (57.7%)	26 (72.3%) *	13 (42%) *	
Pain Location				
Donor site	12 (80%)	21 (80.8%)	12 (92.3%)	0.334
Receipt site	1 (6.6%)	0 (0%)	0 (0%)	
Not surgical site	2 (13.4%)	5 (19.2%)	1 (7.7%)	
Pain Pattern				
Constant	0 (0%)	2 (7.7%)	0 (0%)	0.490
Intermittent	15 (100%)	24 (92.3%)	13 (100%)	
Characters				
Pricking	0 (0%)	1 (3.8%)	1 (7.7%)	0.440
Twitch	3 (20%)	5 (19.2%)	2 (15.4%)	
Dullness	0 (0%)	1 (3.8%)	1 (7.7%)	
Dysesthesia	6 (40%)	13 (50%)	7 (53.8%)	
Numb	6 (40%)	6 (23.2%)	2 (15.4%)	

* Significant difference in patients with spontaneous pain between the mid- and late-recovery stage groups (*p* = 0.019).

**Table 6:** The number of patients that need to be treated or not and its mean NRS values compared with the total mean NRS values at each time interval following anterolateral thigh flap surgery.

Variables	Early (6 m–1 y)	Mid (1–2 y)	Late (>2 y)	*p* Value
	(*n* = 26)	(*n* = 37)	(*n* = 31)	
Pain need to be treated				
No	21 (80.8%)	27 (73.0%)	30 (96.8%)	0.032 *
Yes	5 (19.2%)	10 (27.0%)	1 (3.2%)	
NRS (total)	1.6 ± 1.7	2.1 ± 2.0	0.7 ± 1.0	0.006 *
NRS (be treated)	2.7 ± 1.4	2.9 ± 1.7	1.8 ± 0.8	0.071

Value reveals the mean ± SD, *: *p* < 0.05.

## Data Availability

The data presented in this study are available on request from the corresponding author.
